# Case Report: Clinical Use of a Patient-Individual Magnetic Resonance Imaging-Based Stereotactic Navigation Device for Brain Biopsies in Three Dogs

**DOI:** 10.3389/fvets.2022.876741

**Published:** 2022-07-14

**Authors:** Sarah Gutmann, Thomas Flegel, Marcel Müller, Robert Möbius, Kaspar Matiasek, Florian König, Dirk Winkler, Ronny Grunert

**Affiliations:** ^1^Department for Small Animals, Faculty of Veterinary Medicine, Leipzig University, Leipzig, Germany; ^2^Medical Engineering, Fraunhofer Institute for Machine Tools and Forming Technology, Dresden, Germany; ^3^Department for Neurosurgery, University Hospital Leipzig, Faculty of Medicine, Leipzig, Germany; ^4^Section of Clinical and Comparative Neuropathology, Ludwig-Maximilians-Universität, Munich, Germany; ^5^Small Animal Practice, Neurology, Wiesbaden, Germany

**Keywords:** canine, brain biopsy, 3D printing, veterinary neurosurgery, brain tumor, stereotaxy

## Abstract

Three-dimensional (3D) printing techniques for patient-individual medicine has found its way into veterinary neurosurgery. Because of the high accuracy of 3D printed specific neurosurgical navigation devices, it seems to be a safe and reliable option to use patient-individual constructions for sampling brain tissue. Due to the complexity and vulnerability of the brain a particularly precise and safe procedure is required. In a recent cadaver study a better accuracy for the 3D printed MRI-based patient individual stereotactic brain biopsy device for dogs is determined compared to the accuracies of other biopsy systems which are currently used in veterinary medicine. This case report describes the clinical use of this 3D printed MRI-based patient individual brain biopsy device for brain sampling in three dogs. The system was characterized by a simple handling. Furthermore, it was an effective and reliable tool to gain diagnostic brain biopsy samples in dogs with no significant side effects.

## Introduction

Three-dimensional (3D) printing technology has become an important tool for patient-individual medicine and so has found its way into veterinary neurosurgery in recent years. The technique of 3D printing, also known as additive manufacturing, is used to generate physical patient-individual models from digital files by applying a special material layer for layer (1). In veterinary neurosurgery, 3D printing is used to produce patient-individual anatomical models for surgical planning and education, to design specific neurosurgical devices for diagnostic and treatment purposes of the spine, like patient-specific drill guides for screw placement, and to develop implants for cranioplasty or vertebral fixation (2–14). In addition, 3D printing has been used for brain biopsy procedures in dogs (15–18).

Due to the complexity and vulnerability of the brain, high accuracy and precision during brain biopsy procedures are needed. Those requirements are fulfilled by patient-specific 3D printed navigation devices, and therefore, those constructions are considered to be a safe and reliable option for sampling brain tissues. In a recent cadaver study, the accuracy of the 3D printed magnetic resonance imaging (MRI)-based patient-individual stereotactic brain biopsy frame used in the study presented here was determined (17). A total median target point deviation of 0.83 mm was achieved in that study, therefore reaching a better application accuracy than most brain biopsy systems currently used in veterinary medicine, which have mean needle placement errors ranging from 0.9 to 4.3 mm (19–24) and median needle placement errors of 1.5 mm up to 2.8 mm in dogs (18, 25, 26).

This case report describes the clinical use of an MRI-based patient-individual stereotactic brain biopsy device for brain biopsy procedure in three dogs that have been described in detail elsewhere (16, 17). Intraoperative and postoperative complications, histopathological diagnoses, and neurological outcomes were reported.

## Case Presentation

The first dog, which underwent a brain biopsy procedure with the patient-individual MRI-based stereotactic brain biopsy frame, was a 6-year-old male mixed breed dog. The MRI scan of the brain performed at the referring veterinarian revealed multifocal brain lesions within the cerebrum. Therefore, the dog was referred for a brain biopsy procedure in order to determine the underlying pathology. The second dog was an 8-year-old female boxer. The MRI examination of its brain revealed a mass lesion in the left frontal lobe. The third dog was a 5-year-old male malinois, in which the MRI of the brain showed a small T2-weighted (T2W) and fluid-attenuated inversion recovery (FLAIR) hyperintense lesion in the left ectomarginal gyrus (diameter 3 mm) with no contrast enhancement and bilaterally symmetrical T2W hyperintensities within the hippocampus and the piriforme lobe. All three dogs were presented to the local vet because of generalized tonic-clonic epileptic seizures. Table 1 summarizes the details of the neurological examination, initial MRI findings, further diagnostic tests and the medication prior to brain biopsy, the number of brain biopsy samples, the MRI findings immediately following the biopsy procedure, the days until discharge, the histopathological diagnoses, the MRI findings post brain biopsy, and the adjustments of treatments based on the histopathological diagnoses for all three dogs.

**Table 1:** Results of neurological examination before the brain biopsy procedure, diagnostic tests including CSF analysis and MRI findings, medication prior to brain biopsy, the number of the biopsy samples taken, histopathological diagnoses, MRI findings post brain biopsy, complications, duration of hospitalization, and further treatment for each dog.

**Dog**	**Neurological findings before brain biopsy and neuroanatomical localization (NAL)**	Diagnostic tests inclusive CSF (cisternal)^*^	**MRI findings before brain biopsy**	**Medication prior to brain biopsy**	**Number of taken brain biopsy samples**	**Histopathological diagnosis**	**MRI findings post brain biopsy**	**Complications**	**Days until dis-charge**	**Further treatment**
Dog 1: Mixed breed, male, 6 years, 27 kg	Acute generalized tonic-clonic epileptic seizures, mild generalized ataxia, absent proprioception on the left side, reduced left-sided menace response NAL: forebrain	CBC and blood chemistry unremarkable; x-ray of the chest and ultrasound of the abdomen unremarkable, CSF within normal limits; Toxoplasma gondi, Neospora canium, distemper virus, Anaplasma phagocytophilum-PCR: all negative	Multifocal T2W- hyperintense lesions in the right piriforme lobe, the left marginal and ectomarginal gyrus or gyri, and the occipital lobe part of the right parahippocampal gyrus, affecting both the gray and white matter, T1W predominantly hypointense	Phenobarbital (3.7 mg/kg BID), levetiracetam (37 mg/kg TID), prednisolone (1.3 mg/kg SID)	3	Undefined low-grade glioma/gliomatosis type	T2W-hypointense biopsy trajectory from the brain surface into the center of the T2W-hyperintense lesion in the left marginal gyrus	None	2	Lomustine (90 mg/m^2^ every 4 weeks, later every 6 weeks), radiation therapy (10 x 3Gy whole brain), antiseizure drugs (phenbarbital, levetiracetam, prednisolone in tapering doses after radiation therapy, pregabalin (3.1 mg/kg TID))
Dog 2: Boxer, female, 8 years, 31 kg	Acute generalized tonic-clonic seizures, cluster seizures, reduced proprioception in both hindlimbs NAL: forebrain	CBC, blood chemistry and urinalysis unremarkable; serum antibody titers for Toxoplasma gondi, Neospora canium, and *Borrelia sp*.: negative; CSF: normal NCC and protein, Pandy reaction +, mono-nuclear cells	T2W-hyperintense, T1W-hypointense mass lesion in the left frontal lobe (1.6 x 1.6 x 2.3 cm), mild ring-like contrast-enhancement, mild midline shift to the right	Phenobarbital (3.2 mg/kg TID), levetiracetam (48 mg/kg TID) and potassium bromide (21 mg/kg BID)	2	High grade/anaplastic oligodendroglioma	T2W-hypointense biopsy trajectory from the left frontal sinus into the center of the T2W-hyperintense lesion in the left frontal lobe	None	2	Lomustine (80 mg/m^2^ every 4 weeks), antiseizure drugs (phenobarbital, levetiracetam, potassium bromide)
Dog 3: Malinois, male, 5 years, 34 kg	Progressive refractory generalized tonic-clonic epileptic seizures for 3 years, severe cluster seizures, mildly reduced mental status, generalized ataxia, ambulatory tetraparesis, generalized proprioceptive deficits, absent menace response bilaterally, mild vertically provoked nystagmus NAL: multifocal (forebrain, brain stem)	CBC and blood chemistry: unremarkable, MDR1-defect: genotype free, CSF: unremarkable, urinalysis metabolic screening: unremarkable pattern	Small T2W and flair hyperintense lesions in the left ectomarginal gyrus (diameter 3 mm) and both caudale sylvian gyri, no contrast enhancement, symmetrical T2W hyperintensities (presumptive postictal edema) within the hippocampus, the thalamus, and the piriforme lobe (more severe on the left than on the right)	Phenobarbital (4.4 mg/kg TID), Potassium bromide (21 mg/kg TID), Topiramate (5.9 mg/kg TID), pregabalin (2.2 mg/kg TID)	2	Lymphocytic encephalitis and vasculitis, focal, subacute, mild; CD3 and IBa1 showed a mild increase in positive cells; PCR for canine distemper virus, FSME, Toxoplasma gondii and Neospora canium: negative	T2W-hypointense biopsy trajectory from the brain surface into the T2W-hyperintense lesion in the left ectomarginal gyrus	Non-ambulatory tetraparesis till day 5 after brain biopsy, mild seroma of the biopsy wound	6	Azathioprine (initial 2 mg/kg SID, than EOD), antiseizure drugs (phenobarbital, potassium bromide, topiramate, pregabalin)

* *CSF reference values (cisternal): NCC, nucleated cell count < 6 cells/μl; protein ≤ 0.25 g/l*.

**Figure 1:**
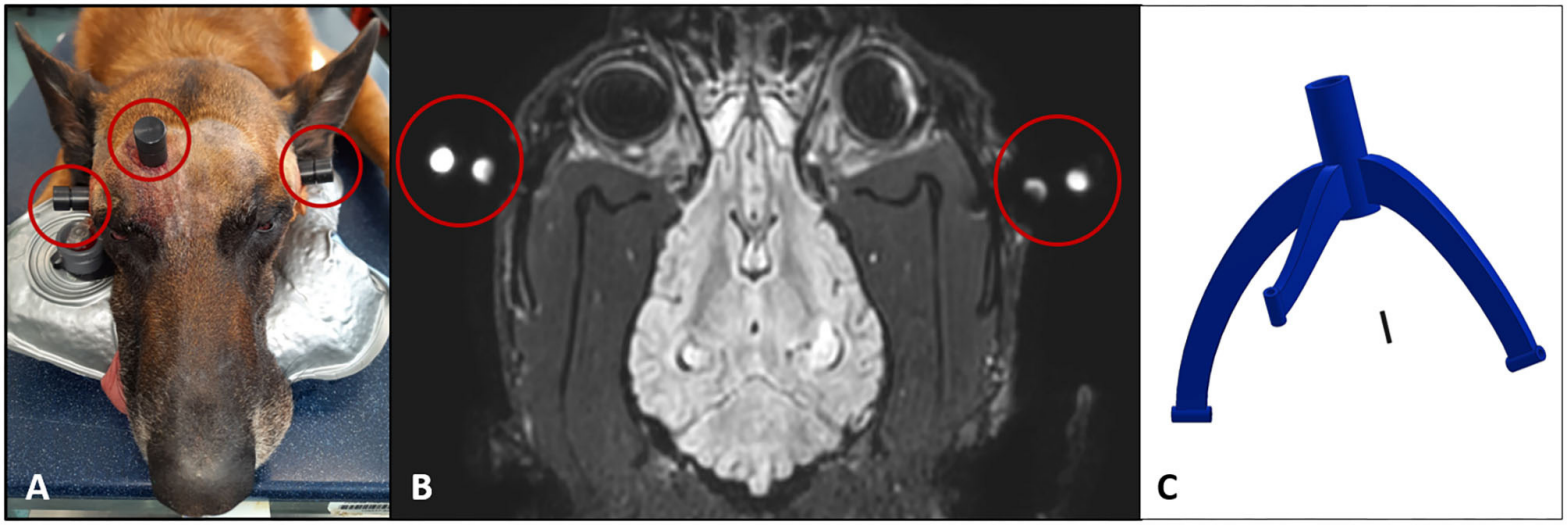
**(A)** Preparation of the patient for the planning MRI scan. Dog head (case 3) with the three bone anchors and specific MRI markers in place at both zygomatic arches and paramedian over the frontal sinus (red circles). **(B)** Dorsal T1W MR image of the planning MRI scan for dog 1 with the MRI markers in place (red circles). The MRI markers were made of small plastic that contained two round press fit vitamin D capsules, which were visible in the MR images. **(C)** The computer-aided construction of the patient-individual brain biopsy device on the basis of the MR images was made by an engineer. The biopsy frame consists of three legs and a biopsy port in prolongation to the biopsy trajectory to the target point in the intracranial lesion.

Before the brain biopsy procedure, all dogs received a planned MRI scan of the head for preparation of the patient-individual stereotactic brain biopsy frame. General anesthesia was induced with diazepam 0.5 mg/kg IV, butorphanol 0.38 mg/kg IV, and propofol as needed for intubation, and dogs were maintained on isoflurane inhalation in 100 % oxygen. The hair paramedian over the frontal sinus and on both sides of the zygomatic arches was clipped. After sterile preparation, 10 mm skin incisions were made and three special titanium bone anchors were drilled into the bony points (paramedian over the frontal sinus, both sides of the zygomatic arches) by using a hand drill. The bone anchors were small commercially available titanium self-cutting screws (diameter 2 mm, length 4 mm; Waypoint^TM^, FHC, Bowdoin, USA) with an inner thread in the head of the bone anchors. The head of the bone anchors extended 4.5 mm over the bony surface. Afterward, special MRI markers were secured to the inner thread of the head of the bone anchors (Figure 1). These MRI markers were made of small plastic cylinders (diameter 15 mm, height 17.5 mm) that contained two round press fit vitamin D capsules (Dekristol 20.000 I.E., Mibe GmbH). The base of those cylinders was secured to the bone anchors by a screw that fits into the inner thread of the anchor. Following placement of bone anchors and attached MRI markers, a planned MRI scan of the head using a slice thickness of 1 mm was performed in sternal recumbency using a head coil with a 3-Tesla MRI (Ingenia, Philipps Healthcare, Hamburg, Germany). Whether a T1W or T2W sequence was used depended on the imaging characteristics of the intracranial lesion. After the MRI scan, the markers were unscrewed from the bone anchors and the skin incisions were closed covering the anchors with a single suture. The dogs were recovered from anesthesia and discharged with a neck collar until the biopsy procedure was performed a few days later. The neck collar was necessary to reduce the risk of loosening the bone anchors by scratching the head. For planning and construction of the biopsy device, the anticipated trajectories to the brain lesions were drawn into the planning MRI scans (case 1: trajectory into the brain lesion in the marginal gyrus of the left cerebrum, case 2: trajectory into the mass lesion of the left frontal lobe, case 3: trajectory into the lesion into the left ectomarginal gyrus). Care was taken to avoid penetration of blood vessels and important neurological structures. The entry point on the brain surface was chosen in the middle of the gyrus. The computer-aided construction and manufacturing of the biopsy devices were made by an engineer as previously described (16, 17). The 3D frames consisted of a biopsy port and three legs each resting on one bone anchor. All devices were made from polyamide material (PA12) by using Multi Jet Fusion (MJF) techniques of the company HP (Jet Fusion 3D 4200, Palo Alto, California, USA). The frames were sterilized with gas sterilization (formaldehyde 3%, 6 h by 60°C).

**Figure 2:**
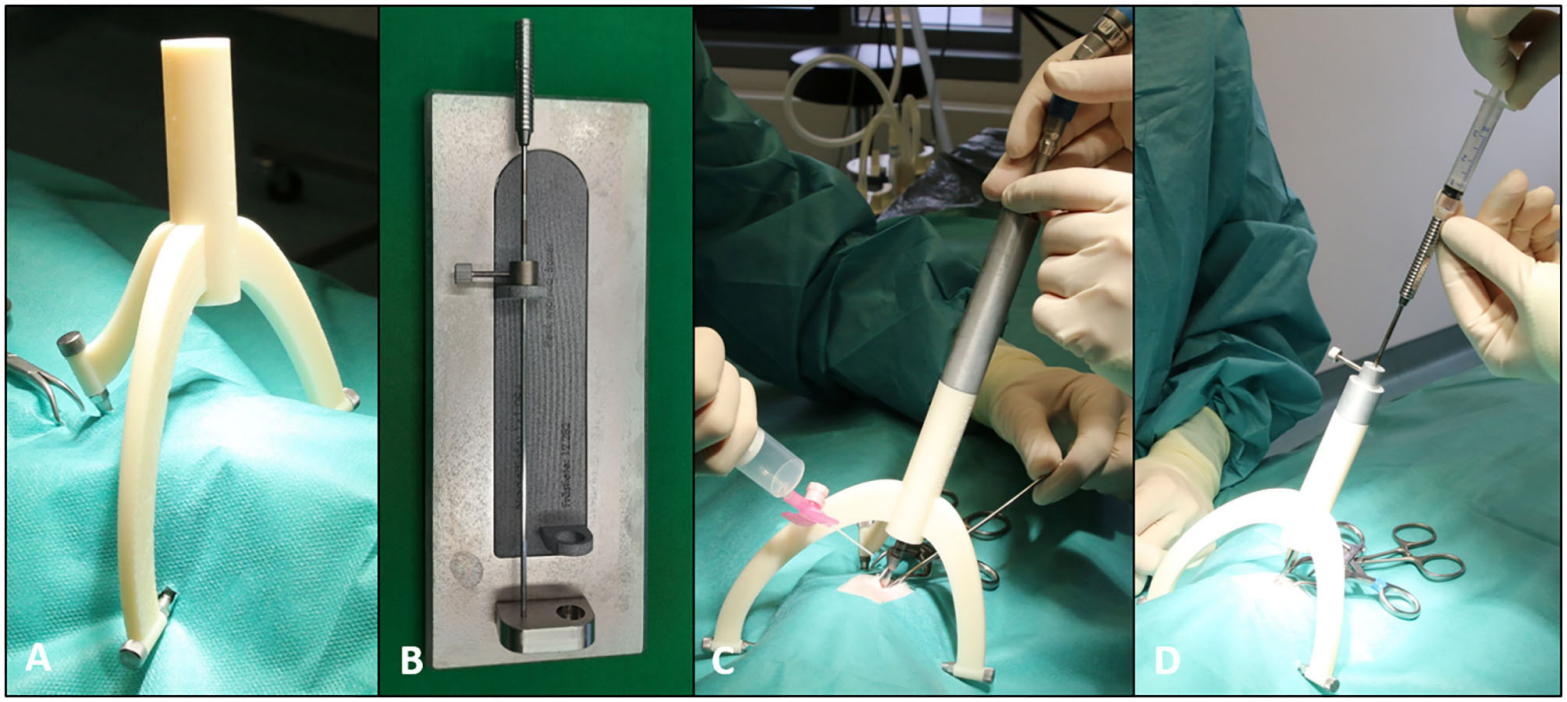
A brain biopsy procedure with the MRI-based patient-individual brain biopsy device. **(A)** A 3D printed biopsy frame (consisting of three legs and a biopsy port) attached to the bone anchors by using specific screws. **(B)** Patient-individual 3D printed measuring instrument for adjustment of the desired depth of the brain biopsy needle with help of a spacer. **(C)** A biopsy frame with the drill sleeve in place during the creation of a 3 mm mini burr hole for brain biopsy. **(D)** A brain biopsy needle placed in needle sleeve with an attached syringe.

The biopsy procedure in cases 1 and 2 was performed 6 days after the planning procedure, whereas in case 3, the biopsy procedure was carried out 2 days after the planning MRI. For those procedures, the dogs received general anesthesia (induced with diazepam 0.5 mg/kg IV, fentanyl 5 μg/kg IV, and propofol as needed for intubation). It was maintained with isoflurane in 100% oxygen (dog 1) or propofol-CRI (dog 2 and 3, 0.2 mg/kg/min IV) in combination with fentanyl-CRI (at 5–12 μg/kg/h IV), cephazolin (30 mg/kg IV), and metamizole (40 mg/kg IV). The hair of the head was clipped between the following borders: craniocaudal: eyebrows to the neck and laterolateral: between both zygomatic arches. The sutures over the three bone anchors were removed and the heads were prepared aseptically. The three legs of the patient-individual 3D biopsy frames were rigidly attached to the bone anchors using three specific screws (Figure 2A). Then, minimally invasive access to the skull was created in the prolongation of the biopsy port. A skin incision of 20 mm was made and the temporal muscle was separated from the skull surface. After placing a drill sleeve into the tool guide of the biopsy port, a round craniotomy of 3 mm was drilled (Electric Pen Drive, DePuy Synthes, West Chester, Pennsylvania, USA) (Figure 2C). Because of the special position of the intracranial lesion in case 2 (left-sided rostral frontal lobe), the access to the brain was performed through the frontal sinus and a two-stage drilling process was needed in this case: A 3 mm mini burr hole into the left frontal sinus was made using the drill sleeve of the biopsy port; then, this small access was enlarged up to 15 mm in diameter to recognize possible bleeding in the depth, and later, the thin bony lamella overlaying the frontal lobe was perforated with a 3 mm drill using the drill sleeve of the biopsy port as well.

As the next step, the dura mater was penetrated in all dogs with a 0.3 mm hypodermic needle. The drill sleeve was then removed and the biopsy sleeve was placed into the tool guide of the biopsy port. A Sedan side-cutting brain biopsy needle with an outer diameter of 2.5 mm was used (ELEKTA, Stockholm, Sweden). The desired depth of the biopsy needle was adjusted with the help of the patient individual 3D printed measuring instruments made of the same material as the biopsy device (Figure 2B). Afterward, the biopsy needle was advanced through the needle sleeve of the tool guide into the brain parenchyma to the planned target point. Two (case 2 and 3) to three (case 1) brain biopsy samples were taken using an aspiration method by attaching a 3-ml syringe to the top of the biopsy needle (Figure 2D). The needle was placed into the intracranial lesion with a closed sampling opening at the tip. When the needle reached its pre-planned depth, the needle sampling area was opened by rotating the internal needle part. Brain tissue adjacent to the biopsy needle was aspirated into the opening by a negative pressure generated by retracting the plunger of the syringe to the 0.5 ml point. Then, the biopsy needle was closed again by rotating the inner part of the needle back into the original position and the brain tissue was cut off. The needle was removed from the skull with a closed side opening. The brain biopsy was flushed out of the biopsy needle into an embedding cassette for biopsies using physiological saline. The cassette was marked with the number of consecutive samples and then fixed in 10% buffered formalin. The direction of the side opening of the biopsy needle was slightly changed between the consecutive biopsies.

After the brain biopsy procedure, the 3D biopsy frame, as well as the bone anchors, were removed and the craniotomy opening was closed with bone wax. Then, the overlying tissue was closed into three layers. In dog 2, the burr hole in the bone overlying the frontal lobe was closed with bone wax and the outer craniotomy was closed using a titanium reconstruction mesh (20 by 30 mm in size) and three small titanium screws. The skin over the three bony points was closed with a single suture.

**Figure 3:**
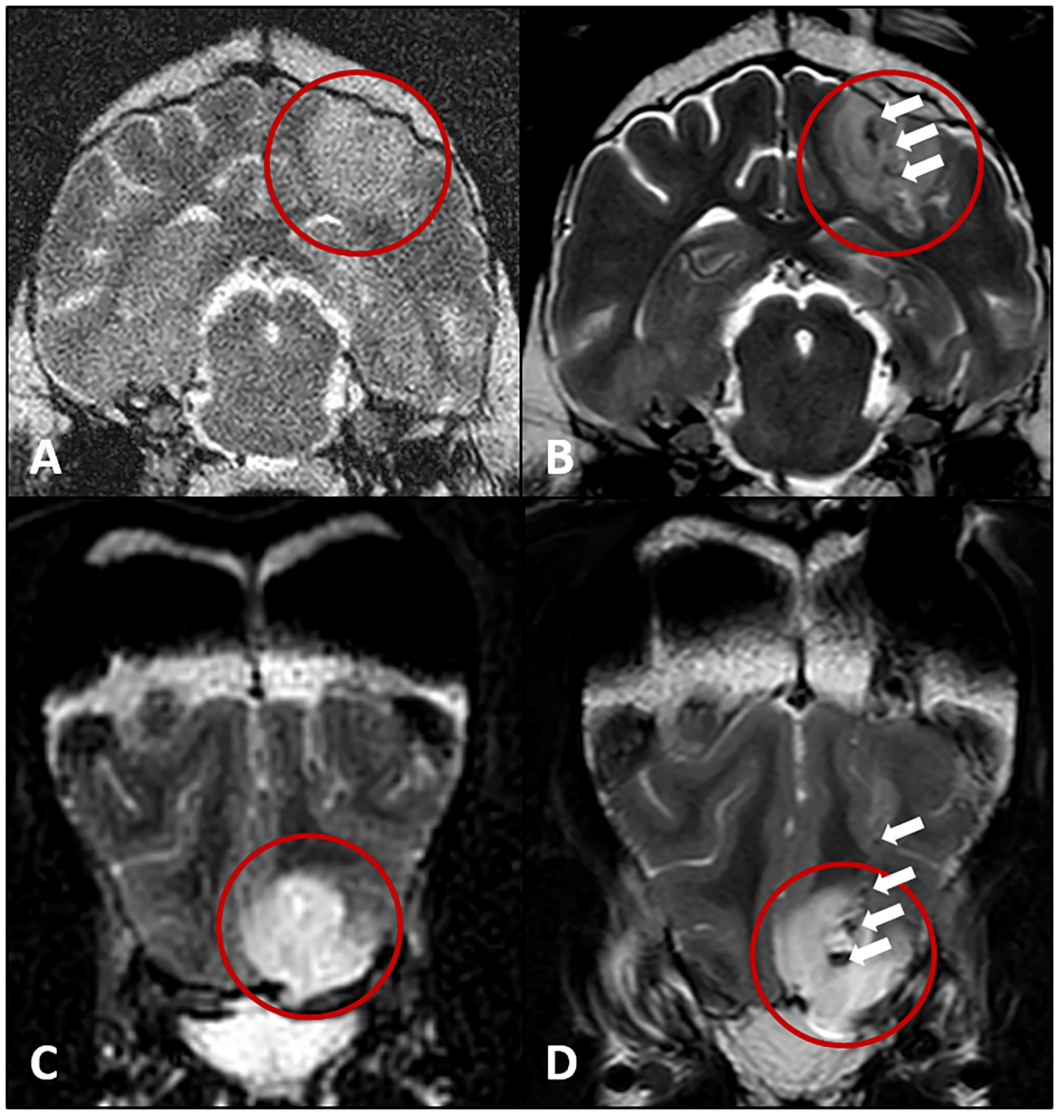
Transverse T2W MRI images of the hyperintense intracranial lesions (red circles) of the dogs in cases 1 and 2 before **(A,C)** and after the brain biopsy procedure **(B,D)**. T2W hypointense artifacts along the biopsy trajectories (white arrows, presumptive air) leading into the brain lesion of cases 1 **(B)** and 2 **(D)**.

All dogs received a control MRI examination of the head immediately following the brain biopsy (case 1 and 2: Figure 3), where a T2W hypointense biopsy trajectory could be visualized. The dogs were monitored in the intensive care unit for 24 h and were treated with antiseizure medication (see Table 1), cephazolin (30 mg/kg TID IV), and analgesia (dog 1: butorphanol-CRI (0.1–0.3 mg/kg/h IV; dog 2 and 3: levomethadone 0.25 mg/kg QID SC and robenacoxib 2 mg/kg SID SC).

In cases 1 and 2, no complication was noticed during or immediately following the biopsy. There was no neurological deterioration and therefore both dogs were discharged 48 h after the procedure on antiseizure and antibiotic medications (cephalexin 28 mg/kg BID PO for 5 days). In case 3, the surgeon experienced difficulties advancing the tip of the biopsy needle into the brain. The needle could not be inserted due to a resistance caused by an incomplete incision of the dura mater. Following an extension of the incision into the dura mater, the biopsy needle could be placed without further resistance. Dog 3 showed a deterioration of the neurological status and was not able to walk unassisted 1 day after brain biopsy. This non-ambulatory tetraparesis improved over the next 4 days, so the dog was discharged 6 days after the brain biopsy procedure with an ambulatory tetraparesis similar to the status prior to the brain biopsy.

**Figure 4:**
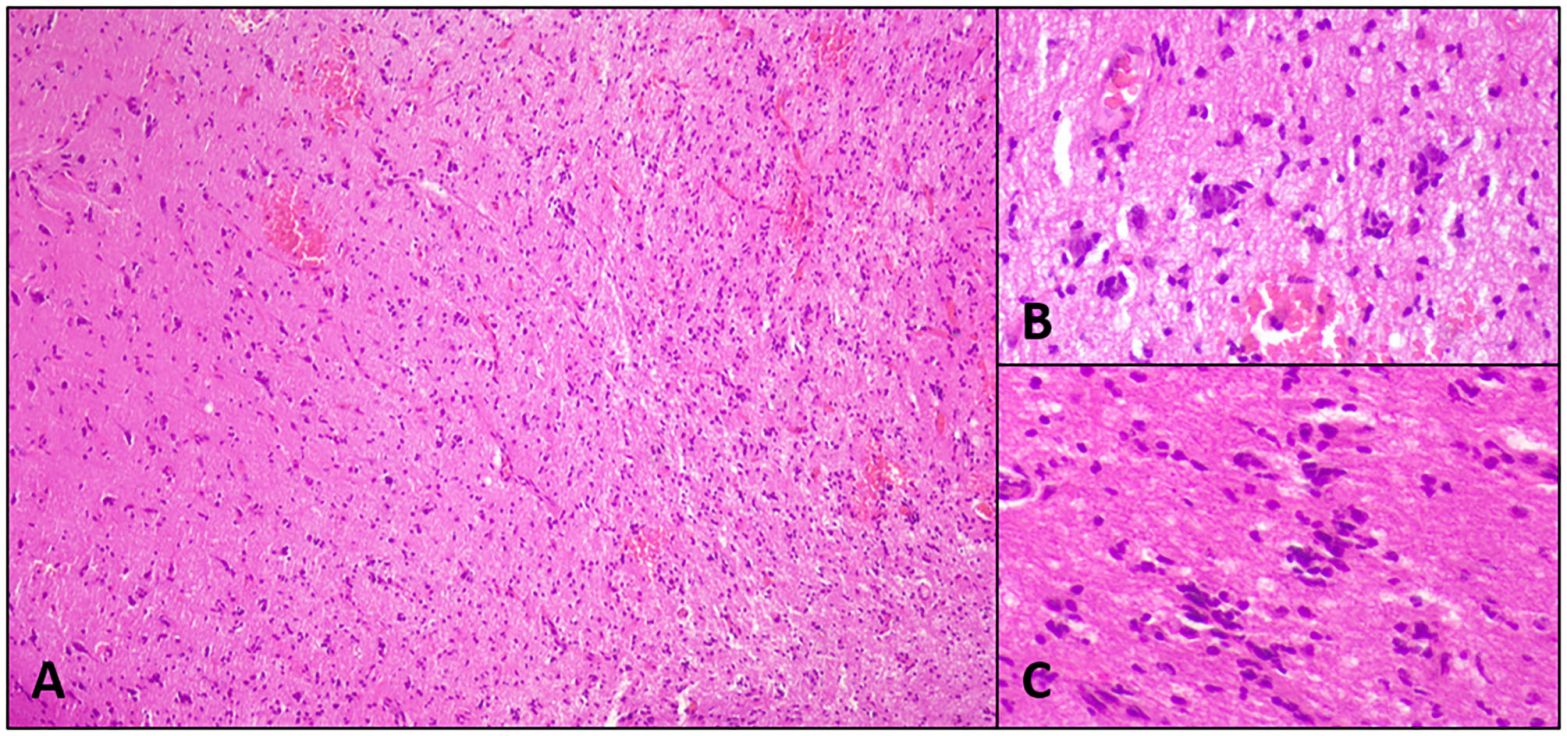
Histopathological images of case 1: Diffuse low-grade spindeloid glioma with sparing of gray matter **(A)**, perineural **(B)**, and perivascular **(C)** structures of Scherer. The cells present with moderate cellular atypia, ovoid to elongated nuclei, stippled chromatin, unremarkable nucleoli, and invisible cell borders. Mitoses are not seen. The cells stained negative for GFAP, MAP2, and OLIG2.

Upon complete fixation, brain samples were routine processed (27). Histopathological analysis followed standard algorithms. In dog 1 the examination of the brain biopsy sample/ biopsy revealed a low-grade neuroglial tumor (glioma) of undefined cellular origin (28), the growth pattern of which on histology and MRI with lesions in the right piriforme lobe, the left parietal lobe, and right occipital lobe was consistent with that of the gliomatosis cerebri histotype (29, 30) (Figures 3A,B, 4). Therefore, in addition to the antiseizure medication (phenobarbital 3.7 mg/kg BID, levetiracetam 37 mg/kg TID, pregabalin 3.1 mg/kg TID), chemotherapy using lomustine (90 mg/m^2^ every 4 weeks) and radiation therapy (10 × 3 Gy, whole brain) were initiated. 1 year later, the dog was presented for a follow-up MRI examination of the brain, where regression of all lesions was visible. The dog was in good general health and had a stable neurologic status. However, the dog had to be euthanized nearly 1.5 years after the brain biopsy procedure because of renal failure potentially caused by lomustine treatment.

In dog 2, the histopathological examination identified high-grade (anaplastic) oligodendroglioma (28), in which former WHO recommendations suggested grade 3 behavior (29). The dog was treated with lomustine (80 mg/m^2^ every 4 weeks) in combination with an antiseizure medication using phenobarbital (3.2 mg/kg TID), levetiracetam (48 mg/kg TID), and potassium bromide (21 mg/kg BID). It was euthanized 11 months after the brain biopsy procedure because of adverse effects due to the chemotherapy.

The histopathological investigation of the brain biopsy samples in dog 3 showed a mild lymphocytic presumptive immune-mediated encephalitis after exclusion of common infectious agents. The dog was treated with antiseizure medication (phenobarbital 4.4 mg/kg TID, potassium bromide 21 mg/kg TID, topiramate 5.9 mg/kg TID, pregabalin 2.2 mg/kg TID) and an immunosuppressive therapy using azathioprine (initially 2 mg/kg SID, followed by the same dose EOD after the first 2 weeks). Those medications did result in significant improvement of the neurological status and reduction in seizure frequency from severe cluster seizures every 2 weeks to three cluster seizures within 6 months. At the time of writing the manuscript, the dog was still alive 6 months after the brain biopsy procedure.

## Discussion

This case report described the clinical use of an MRI-based 3D printed patient-individual stereotactic brain biopsy device. Two of three dogs were diagnosed with specific neoplastic diseases based on histopathological examination of the brain biopsy specimen. In one of those two dogs, the presumptive diagnosis based on MRI examination was an inflammatory disease because of the multifocal distribution pattern. In this case, the diagnosis significantly altered the treatment plan. Therefore, this case report underlines the importance of sampling intracranial lesions for specific histopathological diagnoses. Neither CSF analysis nor brain MRI can reliably differentiate between inflammatory and neoplastic brain disease in veterinary medicine (31–36). Therefore, brain biopsy represents a helpful tool for obtaining specific diagnoses of intracranial lesions.

In both dogs with neoplastic disease, the therapy (chemotherapy with or without radiation) led to an improvement of the dogs' neurological signs for 1 to 1.5 years. The sampling of the lesion of the third dog revealed presumptive immune-mediated encephalitis. The dog showed a significant improvement in immunosuppressive treatment. Due to the small case series, it is not possible to determine a diagnostic yield of the biopsy procedure. In previous literature, the diagnostic yield of other brain biopsy devices was reported to range from 73.9 to 96.4% (15, 25–27, 37–42).

While the study was being conducted, two of the three dogs presented in this case report were already euthanized. Unfortunately, the owners refused a histopathological examination of the dogs' brain postmortem, which is why it is not possible to give a statement on the diagnostic accuracy of the brain biopsy procedure. The diagnostic accuracy indicates whether the histopathological diagnosis determined by brain biopsy is consistent with the results of histopathological examination of the brain postmortem or after partial surgical tumor resection. Few previous studies determined the diagnostic accuracy for brain biopsies, which varied between 81 and 100% (26, 37, 40, 41).

Although diagnostic tissue samples were taken from the brain, they may not necessarily be representative of the primary pathology. Peritumoral inflammation adjacent to a neoplastic lesion or a necrotic area within the neoplastic lesion may have been sampled inadvertently (25). Hence, care must be taken during planning the target point for the stereotactic brain biopsy procedure to avoid such regions. Therefore, the MRI signal behavior of different areas of a lesion, primary vs. secondary changes, should be critically considered.

Dog 1 exhibited temporary bradycardia at the beginning of the anesthesia, which disappeared again without further treatment. Sedation with anesthesia in cases 2 and 3 was uneventful. Two of the three dogs (case 1 and 2) showed no major intraoperative or postoperative complications and no neurological deterioration following the biopsy. Therefore, those dogs could be discharged 48 h after the procedure. The other dog (case 3) showed a temporary worsening of the preexisting tetraparesis and was not able to walk for 4 days following the brain biopsy. This deterioration seems to be associated with a problem experienced by the surgeon during the placement of the biopsy needle, which was probably caused by an incomplete dura mater penetration. The brain may have been intermittently compressed by unsuccessful attempts of needle insertion. The previously reported brain biopsy associated morbidity in dogs ranges from 27 to 30% and mortality rates are reported to be 5.2 to 20% in veterinary literature (25, 27, 37–39, 43).

There are only two other studies describing brain biopsy procedures with the use of 3D printed devices. In one, a 3D printed facemask for brain sampling was used, resulting in specific histopathological diagnoses in four out of five dogs (15). Although no information about the application accuracy of the system is given, it can be assumed that the use of facemasks for brain biopsies is less accurate than the system used here, because the bridge of the nose and the nasal planum were used for the construction of the device, which appears to be problematic, especially in brachycephalic dog breeds. In addition, the face mask cannot be completely rigidly secured to the skull. In the case report presented here, the patient-individual 3D printed frame was rigidly fixed to the skull by using three bone anchors and specific screws, this most likely led to a higher accuracy during brain sampling compared to the use of noninvasive facemasks.

Another study describes the accuracy of a patient-individual 3D printed brain biopsy frame and the use of this system for sampling the brain in two client-owned dogs (18). With that system, one of two dogs could be sampled successfully. One of the disadvantages of this device is that the frame cannot be used with minimal-invasive access because the device is secured directly to the skull surface. A median needle placement error of 2.7 mm (range: 0.86–4.5 mm) is reported (18). The accuracy of the system presented here is determined in a cadaver study with a median target point deviation of 0.83 mm (range: 0.09–2.76 mm; 17). Furthermore, the technique was minimally invasive and all dogs could be sampled successfully in this case report.

The advantages of the patient-individual 3D-printed frames used in the case series presented here are relatively high safety and simple handling, which lead to successful brain biopsy procedures and high accuracy even in deep seeded lesions (16, 17). The frame-based brain biopsy procedure was a relatively short surgical intervention of about 45–60 min. The system can be used in dogs with every size and shape of the skull, because of the patient-individual design. Some other brain biopsy systems have limitations in their use with regard to different skull sizes or shapes, especially in brachycephalic dogs (15, 19, 21). The frame presented in this case report was designed based on MR images only, that is why a computer tomography (CT) scan of the skull was not needed. However, in cases of CT visible intracranial lesions, the same system can be used based on CT images only, because the markers are visible in both imaging modalities. Another advantage of the system presented here is the possibility to sample multiple brain regions in the procedure. The frame can be designed with multiple biopsy ports to sample different lesions in patients with multifocal intracranial disease. Furthermore, in the case of large brain lesions, multiple samples of different areas of the same lesion can be obtained by sampling different depths of the lesion along one trajectory. In the two cases with multifocal lesions (case 1 and 3), just one lesion was sampled. The authors chose the most superficial and easily accessible lesion for planning the biopsy trajectory. In fact, care should be taken to keep the path to the target region short, but care must be taken to ensure that no important neurological structures, blood vessels, or ventricles are penetrated. In this case, a slightly longer path through the brain tissue may be preferable to the shorter path. In order to avoid non-specific findings due to postictal changes, asymmetric lesions would be preferred to symmetrical lesions for sampling. In the future, we recommend the authors to use multiple biopsy ports in cases of multifocal brain lesions if they are accessible. However, care must be taken, because, in human medicine, it is known that an increasing number of brain specimens and multiple biopsy trajectories increase the risk for adverse effects (44, 45).

Another advantage is that the system is less expensive than other rigid stereotactic brain biopsy frames or modern neuronavigation systems.

A major disadvantage of the system was the need to separate the biopsy procedure into two parts, the planning and the actual biopsy, with a few days elapsing between both. Therefore, two general anesthesia were needed. More importantly, the time delay bore the risk of anchor loosening, which made the biopsy procedure using the patient-individual device impossible. This complication was experienced in a fourth dog (dog 0), where another biopsy system had to be used for brain sampling. From that time on, a collar was applied to all dogs between the two procedures in order to prevent scratching or rubbing the dog's head potentially resulting in anchor loosening. Furthermore, anchor loosening had been prevented from that time on. It appears to be crucial to use screws with a large screw head base maximizing the screw bone interface in order to reduce the risk of loosening during the waiting period between screw placement and biopsy procedure since the screws are placed into prominent bony points that are exposed to external forces.

Another disadvantage of the two-step system described here is that patients with a presumably inflammatory intracranial lesion can often not be deprived of anti-inflammatory medication while waiting for the biopsy procedure, whereas using such medication may alter histopathological findings. Therefore, we have adjusted the workflow of designing and printing the biopsy device, resulting in a reduction of the time span between planning and taking the biopsy to 2 days.

The lack of flexibility is another general disadvantage of frame-based stereotactic brain biopsy systems compared to other neuronavigation systems (23, 26, 27), especially when using 3D printed frames, where the target trajectory can no longer be changed. Nevertheless, the information from a well-planned brain biopsy is essential to gain a specific diagnosis and initiate purposeful therapy.

In conclusion, the MRI-based patient-individual 3D printed stereotactic brain biopsy device had simple handling and was an effective and reliable tool to gain diagnostic brain biopsy samples in three dogs.

## Data Availability Statement

The original contributions presented in the study are included in the article/supplementary material, further inquiries can be directed to the corresponding author/s.

## Ethics Statement

Ethical review and approval was not required for the animal study because the study was performed in agreement with the local ethical regulations. All owners of the dogs agreed to the sampling of their dog's brain with the new system and confirmed in writing that the results may be published afterwards. Written informed consent was obtained from the owners for the participation of their animals in this study.

## Author Contributions

SG: acquisition of data, analysis and interpretation of data, and writing the manuscript. TF: conception and design of the study, acquisition of data, and critical revising of the manuscript. MM: conception and design of the biopsy frames and proofreading. RM: 3D print of the biopsy frames, analysis of data, and proofreading. KM: acquisition of data (neuropathology). FK: acquisition of data and proofreading. DW and RG: conception and design of the study and proofreading. All authors contributed to the article and approved the submitted version.

## Funding

This study was supported by a research fund from the GKF (Gesellschaft zur Förderung kynologischer Forschung), project number: 25310896.

## Conflict of Interest

The authors declare that the research was conducted in the absence of any commercial or financial relationships that could be construed as a potential conflict of interest.

## Publisher's Note

All claims expressed in this article are solely those of the authors and do not necessarily represent those of their affiliated organizations, or those of the publisher, the editors and the reviewers. Any product that may be evaluated in this article, or claim that may be made by its manufacturer, is not guaranteed or endorsed by the publisher.
